# Heart Rate Variability and Perceived Stress in Teacher Training: Facing the Reality Shock With Mindfulness?

**DOI:** 10.1177/27536130231176538

**Published:** 2023-05-18

**Authors:** Philipp Beuchel, Colin Cramer

**Affiliations:** 1 Department of Clinical Psychology and Psychotherapy, 9188 University of Tübingen, Tübingen, Germany; 2 Department of School Education, 9188 University of Tübingen, Tübingen, Germany

**Keywords:** stress, teacher training, reality shock, mindfulness, physiological stress, ambulatory assessment

## Abstract

**Background:**

The beginning of the career of teachers is a demanding phase. In the combined roles of teacher and trainee, and in the shift from academic to practical learning, trainee teachers have to acquire competencies in teaching as well as coping with stress. In this phase, the phenomenon of “reality shock” is widely observed.

**Objective:**

A mindfulness training was developed to support teacher trainees during their first year. This intervention study investigated perceived and physiological stress at teachers’ career start and the stress reducing effects of the mindfulness training in this phase.

**Methods:**

In a quasi-experimental design, 19 out of 42 participants from this sample underwent mindfulness-based stress reduction training and a wait-list control group (N = 23) underwent a compact course after post measurements. We measured physiological stress parameters and perceived stress at 3 different time points. Heart rate signals were acquired in ambulatory assessment sequences, including teaching, rest periods, and cognitive tasks. The data were analyzed in linear mixed-effects models.

**Results:**

We found high physiological stress in the very beginning of teacher training, which attenuated over time. The mindfulness intervention only led to a greater reduction in heart rate (*d* = .53 to .74) in situations where the intervention group had shown higher heart rate levels initially, but not in heart rate variability. However, the mindfulness group significantly reduced (*d* = .63) their perceived stress and maintained (*d* = .55) this improvement, while the control group maintained a highly elevated perceived stress level throughout.

**Conclusion:**

The mindfulness training could reduce subjective stress, which otherwise seems to be a long-lasting aspect of beginning teachers’ “reality shock”. Indications of a superior reduction of physiological stress in demanding situations were weak, while generally, undue physiological stress seems to be a temporary phenomenon in the initial phase of teacher induction.

## Introduction

The phenomenon of collapsing expectations when faced with new, underestimated challenges in the beginning of the teaching career—called “reality shock”—is well known.^1^ During teacher training or the first year on the job, young teachers often suffer from increasing stress, which is reflected in decreasing mental health, self-efficacy, and progressive beliefs about teaching, as well as increasing burnout-related attitudes.^2-4^ These beliefs and attitudes can even be associated with early attrition or early retirement from the teaching career, as well as manifest diseases.^5,6^ Furthermore, acute stress is negatively correlated with core executive functions, such as working memory, inhibition, cognitive flexibility and self-monitoring.^7^ These executive functions are associated with teachers’ work performance and students’ motivation and discipline.^8,9^

Despite the indicators of biophysiological pathways between teacher stress and diseases,^10^ little is known about the immediate biophysiological manifestation of stress in this period of teachers’ occupational life. The 2 main biophysiological systems of the stress response—hypothalamic-pituitary-adrenal (HPA) axis and autonomic nervous system (ANS)—are highly coordinated and physically interconnected.^11^ Dysregulations in these systems caused by persistent psychosocial stress are associated with poor stress-related cardiovascular responsiveness and vulnerability for stress-related disorders, such as depression and burnout.^12^

It seems, therefore, crucial that teachers maintain a moderate and not permanently increased stress level during their work in class, regarding both their individual health and their job performance. With respect to the psychosocially stressful nature of the teaching profession, the development of stress management skills is desirable for teachers. Mindfulness-based interventions (MBIs) have been successful in reducing stress in teachers.^13^ A recent systematic review^14^ supports a psychophysiological account of mindfulness buffering acute stress that includes alterations of the ANS and HPA axis activation.^15^

### Heart Rate and Heart Rate Variability

Cardiac parameters like heart rate and heart rate variability are reliable indicators of stress and health risks in many ways. Higher heart rate (HR) and lower vagal tone measured by heart rate variability (HRV) during work and at leisure time are indicators of an effort–reward imbalance,^16^ an important framework of occupational stress. HR is considered as both an indicator of psychological stress and an independent health risk factor.^17^ In many longitudinal studies, an increased HR has been associated with a higher risk of cardiovascular diseases (e.g., hypertension and atherosclerosis) and all-cause mortality, independent of other physical conditions and lifestyle factors (e.g., smoking, leisure time, physical activity, etc.).^18^ Clinically relevant HR increases from very moderate 60–70 beats per minute (bpm) are already suggested at each additional 10 bpm^18^ or even 5 bpm.^19^

HRV accounts for the different lengths of succeeding heartbeats and reflects the balance of the ANS.^20^ Heart rhythm is influenced by physical activity, as well as psychological demand. In response to challenging stimuli, the body produces a stress reaction, that is, prepares itself to physically act by accelerating its heartbeat and breath, tensing its muscles, and discharging hormones. Stressors cause the sympathetic part of the ANS to downregulate the HRV via noradrenaline release, while relaxation increases HRV through the parasympathetic activity, including acetylcholine release.^21^ In a moderate dose, a stressful situation is a healthy activation and an absence of the stress response would even present an indication for dysregulation of the biophysiological stress systems. An enduring stress reaction, by contrast, results in serious adverse effects on health, as follows: the activity of the immune system is suppressed, muscular tension can become chronic, and the risk of cardiovascular diseases increases.^22,23^ Teachers could benefit from stress recovery by regaining their low stress level after class, so that their bodies could fulfil their health-maintaining functions of immune defence, digestion, and rejuvenation.

Parasympathetic and sympathetic modulations occur at different paces. These are visible in the different frequency bands within the HR changes.^20^ Available software, such as Kubios^24^ and Artiifact,^25^ is able to decompose the different frequency bands and calculate numerous measures for HRV. Low frequency HRV reflects both sympathetic and parasympathetic activation.^20^ Thus, it cannot be associated with vagal tone and stress directly. In this study, we focus on parasympathetic activation or vagally mediated HRV. A moderate vagal withdrawal under demand—reflecting an adaptive sympathetic activation—has associated with optimal performance on executive functioning among children.^26^ However, increased resting high frequency HRV, reflecting a high parasympathetic activity or vagal tone, is beneficial in many ways: reduced vagal tone is a sign of occupational stress^27^ and risks of cardiovascular diseases and depression.^28^ A high resting HRV appears to be associated with greater behavioural and emotional self-regulation.^29,30^ High HRV buffers the effect of occupational demands of teachers on their emotional exhaustion.^31^ Additionally, during successful emotional regulation tasks, HRV seems to be increased.^30^ Cognitive functioning is also associated with vagal tone; vagally mediated resting HRV is positively correlated with cortical thickness^32^ and functional integration of individual brain regions into the whole brain network.^33^

Age and gender are important factors that influence HR and HRV. In general, elder persons show substantially lower HRV and slightly higher HR at rest. Female persons show significantly higher HR and slightly higher HRV at rest.^34^

### Mindfulness for (Biophysiological) Stress Reduction

Mindfulness is described as an open and receptive awareness including a nonjudgmental attitude that facilitates distancing from stressors and responding differently to them. Despite the exact working mechanisms remaining uncertain, findings on subjective stress reduction seem to be robust. However, findings on biophysiological measures, such as HRV, are scarce and inconclusive to date.^35^

The American Heart Association recommends meditation practice as an “attractive cost-effective adjunct” because of its beneficial effect on cardiovascular risk.^36^ Several mindfulness-based training programs for teachers have shown to be as effective as other MBIs with respect to the reduction of perceived stress and other therapeutic and non-therapeutic outcomes.^13^ To our knowledge, only 1 substantial study has evaluated the effect of an MBI on heart signals in a teacher sample, which involved measuring each teacher’s resting HR in the classroom after school.^37^ Albeit not significant, the difference between the intervention group (IG) and the control group (CG) reached a noticeable effect size of *d* = .40 in favour of the mindfulness practitioners after the mindfulness training.

Several studies on the effects of MBIs on cardiac measures have demonstrated a correlation between mindfulness and HRV, as well as improvements in HR and HRV after mindfulness training sessions.^38^ Study designs included measurements during resting periods, during mindfulness practice periods, and during or directly following stress inductions. In uncontrolled studies among patients with depression after surgery or cancer, measures of resting HRV increased among patients who participated in a mindfulness-based stress reduction (MBSR) program or group therapy, along with a reduction of clinical symptoms, such as depression, anxiety, perceived stress, anger, and sleep disturbance.^39,40^ HR significantly decreased in an MBSR intervention group, which was maintained after 10 months, while monthly mindfulness training sessions were provided.^41^ Participants of a four-week-long mindfulness training exhibited significantly higher HRV during the cognitive challenge of Wechsler Adult Intelligence Scale-IV than persons in the 2 control groups (interaction with a dog and no treatment), signifying a more adaptive response to stress.^42^ Furthermore, meditation practice, in contrast to listening to a mere description of meditation, led to a significantly increased HRV recovery after a frustration induction through a mock pattern recognition task.^43^

These kinds of studies have not yet been conducted among teachers by directly investigating their professional activity. Preliminary studies in small samples of teachers found differences in physiological stress responses (HR and HRV) between workdays and free days,^44,45^ or periods of perceived high and low workloads.^46^ In this study, we combined an ambulatory measurement of heart signals during different activities with a mindfulness intervention during the teacher induction phase, including the period of reality shock at the beginning of teaching.

## Purpose of the Present Study

**Figure 1.**
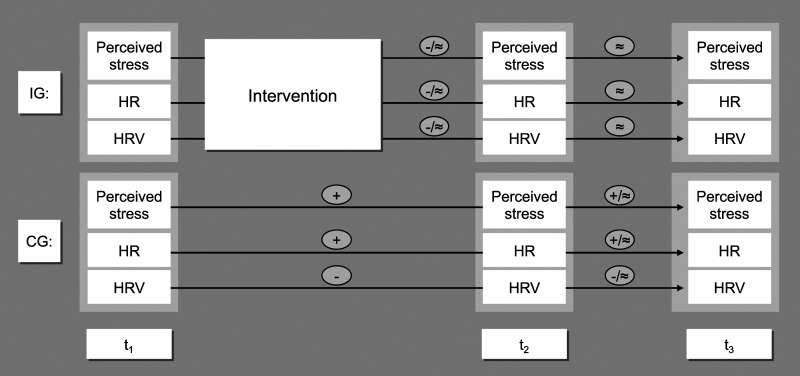
Model of expected changes. *Notes*: IG = intervention group, CG = control group, + = increase, - = decrease, ≈ = no change.

Building on the current state of research, we investigated the effect of a mindfulness-based stress reduction training during the challenging phase of teacher training, and the development of subjectively reported and physiologically measured stress during this period. In another investigation of the same training we had assessed self-reported mindfulness and measures of coping, as well as student perceptions of teaching quality.^47^ There, we had found increased mindfulness and satisfaction with work post intervention, as well as indications for improved classroom management. Now in this study, we expected to find significant differences in physiological markers between active work time and resting time after class at different time points during teacher training (Hypothesis 1). Additionally, we aimed to test the immediate (post) and mid-term (follow-up) effects of the stress-reducing intervention during teacher training. Previous research^2,3,48^ on the German induction phase (for an account of the German two-phased teacher training, see Cortina & Thames)^49^ repeatedly found increasing subjective stress and emotional exhaustion or decreasing psychological well-being during the first year of teacher training, sometimes with a recovery toward the end of the 1.5-year induction phase, resulting in an inverted *U*-shaped development of subjective stress markers. Therefore, we expected increasing stress in the investigated sample during the first year of teacher training, which would translate to higher perceived stress levels and HR, as well as lower vagally mediated HRV at subsequent testing times (Hypothesis 2). Furthermore, we expected a buffering effect on the stress reaction to the reality shock, in the sense of a more favourable development of stress symptoms in the IG. This would mean lower perceived stress, lower HR, and higher vagally mediated HRV, at rest and under strain, compared with the CG that had no stress reducing training at post-intervention, as well as a stabilization of these differences at follow-up compared with a CG that had a presumably significantly less or non-effective intervention just after the second measurement (Hypothesis 3; see Figure 1).

## Methods

### Sample

All 42 participants of this study were young teachers in full-time training, working in higher or lower secondary school and studying at the teacher training institute, where the intervention was offered as an elective course—among other electives such as teaching with digital media and bilingual teaching. All study participants joined a mindfulness program, with the aim to improve, among others, their stress resilience. The IG (N = 19) took a 2-month course before t_2_ (post), whereas the CG (N = 23) attended a 2-day course after t_2_. In each of the 3 cohorts and 2 participating institutes, either the 2-month course or the 2-day course was offered according to a predefined research plan. For participants, no choice was possible between the 2 courses to reduce possible self-selection effects. On average, 7 participants (range: 5 to 9) were in 1 training group.

By far, most of the participants were in their end-twenties (*mean* = 28.6, SD = 4.4). The majority (69.0%) of the participants were female. With respect to gender, IG and CG did not differ from each other (*χ*^*2*^ = .17, *P* = .68) nor from the average (68.7% across all school types; see)^50^ in this occupational group in Germany. Age proved to be similar (*t* = 1.24, *P* = .23) between the 2 groups as well.

### Mindfulness Intervention

The mindfulness course for trainee teachers was an adaptation of the MBSR program (curriculum see Santorelli et al.)^51^ that aimed to reduce stress, to become aware of reactivity toward stress and to cultivate healthy coping habits. Modifications were made to support the transfer of stress-management skills to the interactive professional activity. These modifications included a stronger emphasis on interactive exercises, such as dyadic meditation, mindful partner movements, and improvisation elements. A certain flexibility in the organization of sessions was also allowed; while a total presence course time of 22 hours and a 2-month span were maintained, sometimes, sessions would take place biweekly and were longer in duration to deal with organizational demands. Among the core practices of the MBSR curriculum,^51^ breath awareness, body scan, yoga, mindful eating, compassion meditation, group sharing, mindful daily activity, home practice (including a mindful diary), and a more intense “mindful day” were included. The CG was trained in mindfulness on 2 consecutive days for a total of 10 hours but without mindfulness home practice, and after the second measurement time point. Due to previous research on the efficacy of short-term stress-management interventions (≤10 hours),^52,53^ the CG’s compact course was expected to reduce stress less effective, leading to a significantly lower impact compared to the IG 4 to 5 months later at t_3_. All training sessions were led by one and the same experienced and certified mindfulness teacher. All participants attended at least 80% of the training sessions.

The extent to which the training participants engaged in home practice was assessed in retrospect at t_2_. With the information of how many of the recommended 6 home practice days per week and how much time of the recommended 30 minutes per day was used, the average daily and weekly home practice time was calculated. On average, participants practised 107 minutes per week (SD = 75.5). On the base of 6 practice days per week, this resulted in 17.8 minutes per day (SD = 12.6, range: 4 to 47 min/d).

### Measures

#### Subjective Stress

To measure subjective stress, the German version of the Perceived Stress Scale (PSS)^54^ was applied. This scale is widely used, and its reliability and validity could be demonstrated in a German population, where subjective stress “was consistently associated with depression, anxiety, fatigue, procrastination and reduced life satisfaction”.^54^ Our sample showed a satisfactory internal consistency reliability of α = .88.

#### Physiological Stress

**Figure 2.**
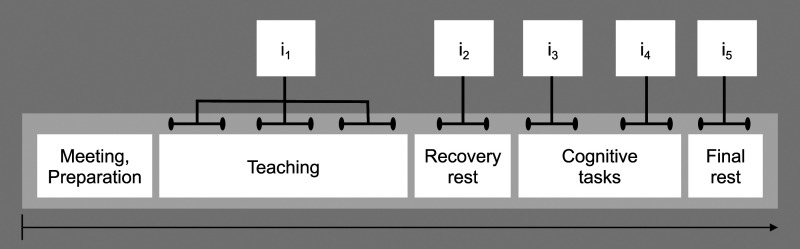
Measurement procedure in school with analysed intervals (i1 to i5).

Physiological stress was measured using a Polar® RS800CX mobile device (including thoracic band, sender, and watch). This device has been used and validated in several studies.^55^ From the recording of a lesson, followed by periods of rest and more or less stressful concentration tests, 5 intervals of interest were selected (Figure 2): (1) teaching, (2) resting after teaching, (3) cognitive test under time pressure (d2-R: marking recognized signs consisting of the letter ‘d’ with 2 strikes, in a row of slightly varying signs),^56^ (4) self-paced cognitive test (INKA: noting down letters behind memorized target letters from a row of letters),^57^ and (5) resting (/baseline value). Each interval lasted for 5 minutes, as recommended by the Task Force of the European Society of Cardiology and the North American Society of Pacing and Electrophysiology.^20^ However, the reported teaching interval data were averaged from the data at 3 single intervals (the beginning, the middle part, and the last third of a lesson). This procedure should ensure a randomization of possibly influential events during class. The teaching interval was different from all the others in that the trainee teachers were changing their posture (standing, walking, and talking), while during resting and cognitive tests they were able to sit and be silent. These differences reduce the comparability between the teaching interval and the 4 other intervals. To increase comparability of the teaching interval between time points, participants were asked to select lessons that were as similarly structured as possible at each of their measurements. In particular, the lesson structure should not differ significantly in the ratio of teacher lecture, group work and silent work of students. During the resting intervals, the researchers did not prompt the participants to practice any of the mindfulness or breathing techniques they had learned, but rather encouraged them to simply relax.

In this study, we measured HR, square root of the mean squared successive differences (RMSSD), and high frequency (HF) power. The latter 2 are recommended as measures of vagal or parasympathetic activation.^20^ HF is considered superior in short term recordings, while RMSSD is more independent of breathing rate. In this way, we included 1 time- and 1 frequency-domain measure among all HRV parameters. As RMSSD and HF were not normally distributed, we normalized them with natural logarithmic transformation,^58^ resulting in lnRMSSD and lnHF as variables (original non-normalized values are available in the Supplementary Materials). Following a reviewer’s suggestion, we also provide LF (low frequency power) and LF/HF ratio values in our Supplementary materials to provide a broader picture of participants’ HRV that includes aspects other than vagally mediated HRV.

For a meaningful statistical analysis, it is essential to use high quality data. To achieve the maximum quality of inter-beat-interval (IBI) data, we processed the intervals, as recommended by Laborde et al.,^58^ and used the Artiifact software^25^ that allows a manual selection based on a visual inspection of artefacts, which are imputed by cubic spline interpolation in the next step. For our data, we found that automatic selection and correction, as implemented by other software (e.g., Kubios), were unsatisfactory. For the calculation of parameters, Artiifact’s default settings^25^ were used, including HF of 0.15–0.4 Hz, Fast Fourier Transformation (FFT), a Hanning window of 256 seconds, and a window overlap of 50%.

### Procedure

Each participant provided written consent to participate in the study. All study procedures were approved by the Federal Ministry of Educational and Cultural Affairs and complied with the Helsinki Declaration.

**Figure 3.**
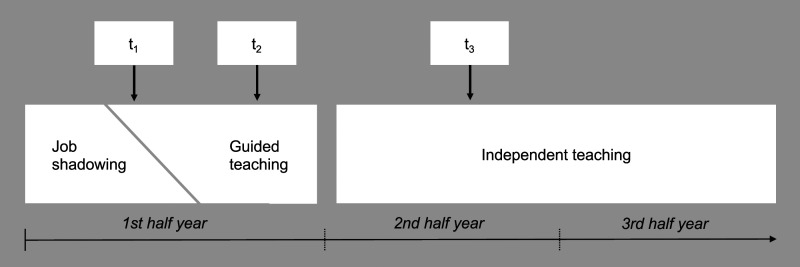
Teacher training (pre-service induction phase), with time points of data collection.

The data were collected at 3 time points—t_1_: just before the mindfulness course for the IG in March/April, t_2_: just after the course in June/July, and t_3_: 5 months afterwards in November/December. Teacher training had started in the beginning of the year. Until the summer holiday (from end-July to mid-September), trainee teachers started by observing other teachers and taught a gradually increasing number of short lessons (6–12 hours) under guidance in different classes. Starting with the new school year in September, they would teach their own classes independently (Figure 3).

The questionnaire was made accessible to participants through a link to the Unipark online survey platform (www.unipark.com). For the data collection in their respective schools, the trainee teachers were asked to select the classes in which they had taught at least twice before the measurement in order to avoid the influence of teaching a totally unknown group of students. Consecutive measurements were planned and executed with maximum similarity regarding the time of day, age group of students, and instructional design.

The testing procedure was as follows: the testing personnel and the trainee teachers met in the schools approximately 30 minutes before classes started. The researchers guided participants in the correct application of Polar® RS800CX thoracic band, sender, and watch. After class, the participants and the testing personnel proceeded to another room, where they could be undisturbed. After the participants sat down, a 6-minute resting period started, followed by the cognitive tests and another 6-minute resting period in the end (Figure 2).

### Data Analysis

The data were checked for pre-differences, with a 2-tailed *t* test for perceived stress and multivariate ANOVAs for the physiological stress measures at each interval separately. We applied mixed-effects models using restricted maximum likelihood estimation^59^ for the analysis of the dependent variables, especially to deal with the unbalanced design caused by missing data. To test whether the different intervals (i1 to i5) produced different physiological stress levels (Hypothesis 1), for each HR parameter, we specified a model with the fixed effects of intervals and with time, intervention, gender, and age (grouped into younger and older participants by the median) as covariates. Additionally, the individual level was specified as random effect accounting for the dependence of several data cells belonging to the same participant across time and intervals. Similarly, cohort and institute were controlled for. Multiple comparisons were Bonferroni-adjusted. For the change over time (Hypothesis 2), we specified models with perceived stress and HR parameters as dependent variables at each interval separately. Fixed effects included time, group, gender, and age. The individual level was added as a random effect again. For the effect of the intervention (Hypothesis 3), we added the time–group interaction to the previous model. Following the suggestion of a reviewer, we additionally carried out analyses of the associations between biophysiological parameters and change in PSS between post and pre (instead of group). In these cases, the PSS change score and—to control for initial levels—pre values replaced time as independent variables. The biophysiological parameters at post were the dependent variables in the linear models. All analyses were carried out in *R*,^60^ particularly the *lme4* package^61^ for linear mixed-effect models. RMSSD and HF were not normally distributed but skewed. Therefore, we performed a natural logarithmic transformation (lnRMSSD, lnHF) as recommended by Laborde et al.^58^ Effect sizes were calculated based on pooled standard deviations and adjusted means. The significance level was set at *P* < .05 in all cases.

## Results

### Quality of Data

The average percentage of artefacts in the IBIs was 1.1% (SD = 2.5). Six intervals (1.0% of all the available intervals) had more than 10% artefacts and were beyond the possibility of meaningful correction. These intervals were discarded from the analysis, as were missing data; 1 participant dropped out after t_1_, and 4 could not provide their HR data at t_3_. One of them had dropped out after t_2_ and therefore did not provide self-reports at t_3_ either, which were also missing from 1 more participant. Thus, data of n = 41 participants and *k* = 585 usable data points entered the analysis of physiological parameters.

### Pre-Differences

The multivariate comparison of all 3 physiological variables at t_1_ revealed marginally significant differences between IG and CG at the teaching interval (i1; *P* = .06) and the task under time pressure (i3; *P* = .07). In particular, the HR comparison reached significance (*P* = .009) at i3, and marginal significance (*P* = .06) at i1, with higher values for the IG. All other variables did not differ significantly between IG and CG before the intervention.

## Distinctness of Intervals

In comparing the intervals at each time point separately (see Table 1), we found a considerable difference between HR and the HRV parameters: overall, HR decreased from teaching to final rest at all 3 time points, with a small increase from recovery rest directly after teaching (i2) to the consecutive task under time pressure (i3) in between, which was largest at t_1_ and disappeared toward t_3_. Analysing the interval differences when controlling for time point, group, gender and age, the teaching interval (i1) stood out with a much higher HR (*P* < .0001 for all comparisons, *d* = −1.34 to −1.73), while the first resting interval (i2) and the test under time pressure (i3) were similar (*P* = .08, *d* = .11), as well as the self-paced test (i4) and the final resting interval (i5) (*P* = .26, *d* = .07). In comparison to i4 and i5, i3 showed highly significant differences (*P* < .0001 for both comparisons, *d* = −.32 and *d* = −.39, respectively), while for i2, these differences were slightly smaller (*P* < .001, *d* = −.21 and *d* = −.28, respectively).

**Table 1.** Descriptive Statistics for Perceived Stress and Physiological Stress Markers at the 5 Intervals: Adjusted Means and SDs.

Interval	Overall			Intervention Group			Control Group		
	t_1_	t_2_	t_3_	t_1_	t_2_	t_3_	t_1_	t_2_	t_3_
PSS									
	2.03 (.62)	1.93 (.62)	1.89 (.67)	2.11 (.64)	1.81 (.52)	1.79 (.72)	1.94 (.61)	2.05 (.68)	1.98 (.64)
HR									
1	108.2 (15.5)	99.6 (12.4)	97.7 (11.8)	112.9 (11.3)	102.8 (12.8)	98.8 (13.4)	103.4 (17.5)	96.5 (11.6)	96.7 (10.7)
2	85.6 (12.9)	79.8 (8.8)	79.5 (9.4)	88.7 (13.2)	82.6 (7.6)	80.3 (9.8)	82.6 (12.2)	76.9 (8.9)	78.6 (9.1)
3	88.5 (14.3)	81.0 (9.4)	79.5 (8.8)	93.7 (14.7)	82.4 (6.9)	80.4 (9.0)	83.3 (11.6)	79.5 (10.9)	78.6 (8.6)
4	81.9 (11.9)	77.5 (10.1)	76.6 (9.4)	84.8 (10.0)	78.9 (7.2)	77.7 (9.4)	79.0 (12.9)	76.1 (12.1)	75.5 (9.5)
5	79.9 (11.2)	76.8 (9.1)	75.2 (8.0)	82.6 (10.2)	78.6 (7.2)	75.4 (8.2)	77.3 (11.7)	75.0 (10.3)	74.9 (8.1)
lnRMSSD									
1	2.76 (.56)	2.96 (.40)	3.04 (.43)	2.69 (.45)	2.94 (.37)	2.98 (.41)	2.83 (.64)	2.98 (.44)	3.11 (.44)
2	3.31 (.73)	3.55 (.55)	3.50 (.58)	3.23 (.63)	3.41 (.49)	3.40 (.49)	3.40 (.81)	3.69 (.55)	3.59 (.64)
3	3.01 (.74)	3.19 (.60)	3.26 (.67)	2.86 (.59)	3.10 (.45)	3.17 (.53)	3.16 (.83)	3.27 (.69)	3.34 (.76)
4	3.27 (.70)	3.37 (.59)	3.39 (.63)	3.17 (.56)	3.30 (.39)	3.25 (.48)	3.37 (.79)	3.43 (.72)	3.52 (.72)
5	3.47 (.66)	3.50 (.58)	3.55 (.63)	3.40 (.59)	3.39 (.47)	3.51 (.56)	3.53 (.71)	3.61 (.65)	3.59 (.69)
lnHF									
1	4.86 (1.10)	5.06 (.84)	5.20 (.88)	4.87 (1.01)	5.02 (.74)	5.04 (.77)	4.86 (1.19)	5.09 (.92)	5.36 (.95)
2	5.69 (1.41)	6.08 (1.14)	5.98 (1.23)	5.64 (1.25)	5.86 (1.10)	5.86 (1.03)	5.74 (1.56)	6.29 (1.13)	6.11 (1.37)
3	4.91 (1.65)	5.04 (1.29)	5.34 (1.32)	4.75 (1.20)	4.93 (.96)	5.26 (1.10)	5.07 (1.97)	5.16 (1.51)	5.42 (1.50)
4	5.66 (1.44)	5.73 (1.21)	5.78 (1.21)	5.55 (1.09)	5.61 (.78)	5.48 (1.20)	5.77 (1.70)	5.86 (1.48)	6.09 (1.27)
5	5.91 (1.32)	5.92 (1.26)	5.99 (1.26)	5.85 (1.23)	5.68 (1.12)	5.92 (1.16)	5.97 (1.41)	6.15 (1.33)	6.06 (1.36)

*Notes*: See Supplementary Materials for non-normalized (non-log-transformed) RMSSD and HF values.

**Figure 4.**
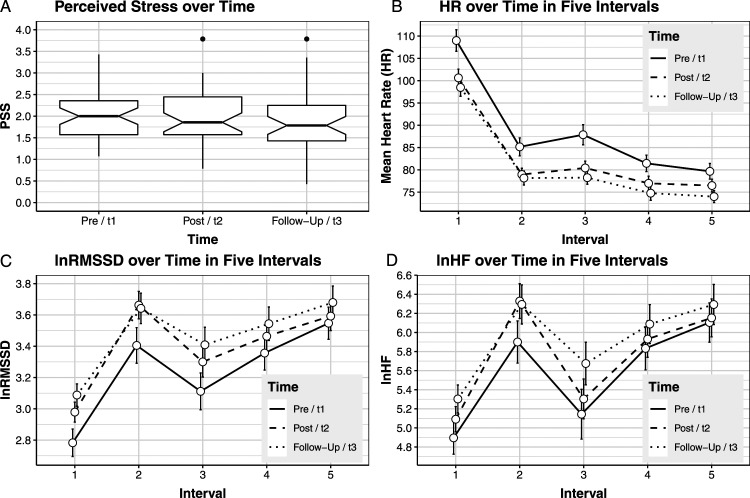
Stress over time in the whole sample. *Notes:* (a) lower and upper box hinges = first and third quartiles; notches = confidence intervals; lines = range; points = outliers; (b-d) Error bars represent standard errors.

In contrast, the HRV parameters showed a pattern of both resting intervals reaching peaks of very similar HRV values (lnRMSSD i2−i5: *P* = .29, *d* = .07; lnHF: *P* = .80, *d* = .02). For lnHF, the self-paced cognitive task was not significantly below that (i2−i4: *P* = .17, *d* = −.16; i4−i5: *P* = .08, *d* = −.17), and the task under time pressure was somewhat closer to the teaching interval (i1−i3: *P* = .03, *d* = .20) than to the other intervals (*P*-values <.0001, *d* = .45 to .62). For lnRMSSD, the self-paced cognitive task was not significantly different from the recovery resting (i2−i4: *P* = .87, *d* = .16) but it was from the final resting (i4−i5: *P* < .01, *d* = .23). The level of the task under time pressure (i3) was between those of the teaching interval and the others, with highly significant differences (*P*-values <.0001, *d* = .29 to .52) from all of them. (cf. Figure 4(b)-(d)).

### General Time Effect

As indicated by the predominantly overlapping confidence intervals (CIs; Figure 4(a)), perceived stress did not change significantly over time across groups (t_2_: *β* = −.08, *t* = −.84, *P* = .40, *d* = −.12, 95% CI [−.25, .10]; t_3_: *β* = −.12, *t* = −1.35, *P* = .18, *d* = −.19, 95% CI [−.30, .05]) and showed a weak tendency to decrease (Table 2).

**Table 2.** General Time Effect Across Groups on Perceived Stress and on Heart Rate Parameters at the Different Intervals.

		Pre to Post (t1−t2)			Pre to Follow-Up (t1−t3)		
Variable	*F*	*t*	*d*	95% CI	*t*	*d*	95% CI
PSS	.93	−.84	−.12	[−.25, .10]	−1.35	−.19	[−.30, .05]
i1 (teaching)							
HR	13.87^***^	−4.15	−.60^***^	[−12.33, −4.43]	−4.81	−.72^***^	[−14.19, −6.00]
lnRMSSD	10.24^***^	3.15	.41^**^	[.07, .32]	4.35	.58^***^	[.16, .41]
lnHF	4.01^*^	1.64	.21	[−.04, .44]	2.81	.37^**^	[.11, .60]
i2 (resting)							
HR	8.60^***^	3.57	−.53^***^	[−9.06, −2.65]	3.54	−.54^***^	[−9.29, −2.69]
lnRMSSD	6.89^**^	3.54	.38^***^	[.11, .37]	2.63	.29^*^	[.05, .32]
lnHF	3.91^*^	2.68	.31^**^	[.11, .69]	1.95	.23	[.00, .60]
i3 (cognitive test under time pressure)							
HR	13.07^***^	−3.98	−.62^***^	[−10.89, −3.68]	−4.65	−.74^***^	[−12.48, −5.10]
lnRMSSD	4.99^**^	2.17	.25^*^	[.02, .32]	3.03	.35^**^	[.09, .39]
lnHF	3.64^*^	.85	.09	[−.17, .44]	2.66	.29^**^	[.11, .74]
i4 (self-paced cognitive test)							
HR	8.61^***^	3.29	−.39^**^	[−6.84, −1.74]	−3.78	−.47^***^	[−7.88, −2.52]
lnRMSSD	1.35	1.24	.15	[−.05, .24]	1.53	.19	[−.03, .28]
lnHF	.38	.48	.06	[−.23, .38]	.87	.11	[−.17, .46]
i5 (resting)							
HR	6.27^**^	−2.42	−.31^*^	[−5.55, −.59]	−3.42	−.46^**^	[−7.12, −1.95]
lnRMSSD	.83	.62	.06	[−.08, .15]	1.28	.13	[−.04, .20]
lnHF	.22	.19	.02	[−.22, .27]	.64	.07	[−.17, .34]

*Notes*: †*P* ≤ .10, *P ≤ .05, **P ≤ .01, ***P ≤ .001; Between-group effect sizes are based on adjusted change scores and pooled *SD*.

Among the physiological data, HR decreased significantly at t_2_ and t_3_, with medium (i1, i2, i3) to small (i4, i5) effect sizes (Figure 4(b); Table 2). HRV parameters increased significantly over time at i1, i2, and i3, with mostly small effect sizes. At i4 and i5, HRV improved consistently but not significantly (Figure 4(c)-(d); Table 2).

### Intervention Effect

A significant time–group interaction in favour of the IG was found for perceived stress between t_1_ and t_2_ (*t* = −2.30, *P* = .02, *d* = −.63). This interaction remained significant at the 5-month follow-up (t_3_), relative to t_1_ (*t* = −1.99, *P* = .05, *d* = −.55).

Significant time–group interactions in favour of the IG were found for HR at the task under time pressure (i3; t_2_: *t* = −2.09, *P* = .04, *d* = −.62; t_3_: *t* = −2.34, *P* = .02, *d* = −.74) and a marginally significant difference at t_3_ at the teaching interval (i1; *t* = −1.82, *P* = .08, *d* = −.53) and the final resting interval (i5; *t* = −1.67, *P* = .07, *d* = −.49). However, in all cases, the IG reduced its higher values from t_1_ to a level similar to that of the CG. Both HRV parameters did not show any significant time–group interaction (Table 3).

**Table 3.** Intervention (Time–Group Interaction) Effects on Perceived Stress and on Heart Rate Parameters at the Different Intervals.

		Pre to Post (t1−t2)			Pre to Follow-Up (t1−t3)		
Variable	*F*	*t*	*d*	95% CI	*t*	*d*	95% CI
PSS	3.14^*^	−2.30	−.63^*^	[−.74, −.06]	−1.99	−.55^*^	[−.69, −.01]
i1 (teaching)							
HR	1.59	−.78	−.23	[−11.06, 4.47]	−1.82	−.53^†^	[−15.49, .62]
lnRMSSD	.40	.83	.22	[−.14, .35]	.09	.02	[−.24, .26]
lnHF	.93	−.31	−.08	[−.55, .40]	−1.31	−.35	[−.83, .16]
i2 (resting)							
HR	1.01	−.31	−.04	[−6.74, 5.91]	−1.31	−.40	[−10.91, 2.13]
lnRMSSD	.37	−.81	−.03	[−.38, .15]	−.13	−.18	[−.29, .25]
lnHF	.64	−1.13	−.27	[−.92, .24]	−.51	−.12	[−.76, .43]
i3 (cognitive test under time pressure)							
HR	3.36^*^	−2.09	−.62^*^	[−14.37, −.57]	−2.34	−.74^*^	[−15.60, −1.49]
lnRMSSD	.49	.88	.20	[−.16, .44]	.82	.19	[−.18, .44]
lnHF	.11	.29	.06	[−.52, .71]	.46	.10	[−.48, .78]
i4 (self-paced cognitive test)							
HR	1.02	−1.15	−.28	[−8.06, 2.04]	−1.30	−.33	[−8.83, 1.75]
lnRMSSD	.36	.40	.10	[−.23, .36]	−.47	−.12	[−.38, .23]
lnHF	.86	−.07	−.02	[−.62, .58]	−1.18	−.30	[−1.02, .24]
i5 (resting)							
HR	1.71	−.83	−.17	[−6.57, 3.18]	−1.67	−.49^†^	[−9.87, .27]
lnRMSSD	.55	−.65	−.13	[−.31, .15]	.41	.08	[−.19, .29]
lnHF	1.22	−1.42	−.28	[−.84, .13]	−.10	−.02	[−.53, .48]

*Notes*: †*P* ≤ .10, *P ≤ .05; Between-group effect sizes are based on adjusted change scores and pooled SD; negative leading signs indicate a larger reduction in the intervention group.

### Effect of Change in Perceived Stress

The change of perceived stress from pre to post was not associated with biophysiological parameters after the intervention, controlling for initial levels (Table 4). We found neither a statistically significant association (all *P* values >, 1), nor an at least small effect (all effect sizes *d* < .20 or > −.20).

**Table 4.** Effects of Change in Perceived Stress From Pre to Post on Heart Rate Parameters at the Different Intervals.

	Post (t2)		
Variable	*t*	*d*	95% CI
i1 (teaching)			
HR	−.23	−.04	[−.35, .28]
lnRMSSD	−.13	−.02	[−.29, .26]
lnHF	.06	.01	[−.27, .28]
i2 (resting)			
HR	−.10	−.02	[−.34, .31]
lnRMSSD	−.45	−.05	[−.28, .18]
lnHF	−.20	−.02	[−.25, .21]
i3 (cognitive test under time pressure)			
HR	.94	−.17	[−.53, .20]
lnRMSSD	.20	.03	[−.27, .33]
lnHF	−.35	−.04	[−.30, .21]
i4 (self-paced cognitive test)			
HR	−.45	−.06	[−.35, .22]
lnRMSSD	.33	.04	[−.21, .29]
lnHF	.02	.00	[−.25, .25]
i5 (resting)			
HR	.04	.01	[−.30, .31]
lnRMSSD	−.37	−.04	[−.27, .19]
lnHF	−.37	−.04	[−.28, .20]

*Notes*: negative leading signs indicate reduction of the variable value associated with reduction of perceived stress from pre to post.

### Influence of Age and Gender

The perceived stress was evidently higher among older participants and females. These medium-sized effects (age: *d* = .48; gender: *d* = .33) did not reach statistical significance. Age did not significantly influence any of the physiological outcomes (all effect sizes *d* < .30), with a tendency for lower HR and HRV in the group of older participants (Table 5).

**Table 5.** Age and Gender Effects.

	Interval	Mean (*SD*)		Between-Group Comparison		
Age		Younger	Older	*t*	*d*	95% CI
PSS		1.80 (.54)	2.10 (.74)	1.55	.48	[−.07, .68]
HR	1	103.1 (13.7)	100.4 (14.1)	−.70	−.19	[−10.08, 4.64]
	2	81.7 (10.5)	81.6 (11.7)	−.01	.00	[−6.14, 6.06]
	3	84.2 (11.5)	81.8 (12.4)	−.73	−.20	[−8.73, 3.87]
	4	78.9 (11.1)	78.4 (10.7)	−.14	−.04	[−7.19, 6.23]
	5	77.6 (10.1)	77.1 (9.4)	−.16	−.05	[−6.46, 5.47]
lnRMSSD	1	2.91 (.54)	2.93 (.40)	.15	.05	[−.27, .31]
	2	3.51 (.62)	3.39 (.63)	−.58	−.18	[−.49, .26]
	3	3.18 (.68)	3.12 (.67)	−.28	−.09	[−.46, .34]
	4	3.43 (.62)	3.25 (.64)	−.93	−.29	[−.57, .20]
	5	3.57 (.63)	3.44 (.59)	−.68	−.22	[−.53, .25]
lnHF	1	5.04 (1.04)	5.05 (.83)	.03	.01	[−.59, .61]
	2	6.04 (1.28)	5.80 (1.19)	−.63	−.19	[−.98, .49]
	3	5.12 (1.43)	5.08 (1.44)	−.08	−.03	[−.93, .85]
	4	5.90 (1.25)	5.55 (1.29)	−.86	−.27	[−1.11, .42]
	5	6.05 (1.23)	5.82 (1.28)	−.56	−.18	[−1.00, .55]
Gender		Female	Male			
PSS		2.05 (.63)	1.84 (.66)	.98	.33	[−.20, .61]
HR	1	104.2 (14.5)	99.5 (12.4)	1.10	.33	[−3.40, 12.70]
	2	81.0 (11.2)	82.3 (10.3)	−.37	−.12	[−7.95, 5.37]
	3	82.4 (12.5)	83.6 (10.2)	−.34	−.10	[−8.11, 5.66]
	4	78.0 (11.5)	79.4 (9.1)	−.36	−.13	[−8.73, 5.94]
	5	77.0 (10.2)	77.6 (8.8)	−.16	−.06	[−7.06, 5.97]
lnRMSSD	1	2.97 (.52)	2.88 (.38)	.55	.19	[−.23, .41]
	2	3.64 (.63)	3.27 (.48)	1.74	.59^†^	[−.03, .78]
	3	3.34 (.69)	2.96 (.52)	1.62	.55	[−.07, .82]
	4	3.50 (.63)	3.19 (.57)	1.41	.48	[−.11, .73]
	5	3.66 (.61)	3.35 (.57)	1.39	.50	[−.12, .73]
lnHF	1	5.12 (1.00)	4.96 (1.81)	.48	.17	[−.49, .82]
	2	6.36 (1.23)	5.48 (1.01)	2.10	.69^*^	[.08, 1.68]
	3	5.54 (1.40)	4.65 (1.29)	1.76	.62^†^	[−.08, 1.87]
	4	6.05 (1.26)	5.40 (1.13)	1.49	.51	[−.18, 1.49]
	5	6.35 (1.21)	5.52 (1.13)	1.87	.65^†^	[−.02, 1.68]

*Notes*: Younger stands for participants aged <28 years (= median); †*P* ≤ .10, *P ≤ .05; Between-group effect sizes are based on adjusted change scores and pooled SD; negative leading signs indicate a larger value for older or male participants respectively.

Gender had an influence on HRV parameters. At all but the teaching interval (i2 to i5), lnHF reached medium-sized effects (*P* < .05 in one case; *P* < .1 in two cases) favouring the female participants but not at the teaching interval (i1). Similarly, lnRMSSD reached effect sizes around *d* = .50 at all but the teaching interval, though the models showed a less significant influence (*P* < .1 in 1 case), and at the teaching interval, the effect was again very small. The HR was slightly elevated (+4.65 bpm; *d* = .33, *P* = .28) among female trainee teachers at the teaching interval only. Other than that, it lay slightly below the level of the male participants (around −1 bpm; *d* = −.13 to −.06, *P*-values >.70).

## Discussion

In this study, we aimed to shed light on the occupational stress of young teachers in their induction phase. The data were obtained from subjective self-report of stress, which is reportedly a potential predecessor of burnout, early attrition, and poor job performance, as well as from physiological markers. The latter markers added another, more objective perspective on stress and were reported as potential indicators of stress-related diseases.

In the data collection, we aimed to be as ecologically valid as possible, with ambulatory measurements during a real occupational activity and concentration tests being similar to class preparation or correction work that has to be done in school (cf. the similarity between a cognitive task and seated work of teachers, as reported by Steptoe et al.).^62^ The specificity of selected activities assumed in Hypothesis 1 was shown by the teaching interval (i1) being significantly different from both resting intervals (i2 and i5) for all physiological measures. The stress-influenced levels of physiological markers during teaching are not unequivocally unhealthy or a sign of distress. They include a functional component of adapting to more physical activity (walking, standing, talking) and readiness to take decisive action in conflicts. Similarly, the interval of the task under time pressure (i3) was different from all other intervals with respect to HRV, indicating a functional activation of the stress response. Only HR was similar at i3 to the not yet attenuated level of the recovery interval (i2) directly after teaching.

While a part of the difference in HRV between i1 and the other intervals could be attributed to the difference in position (standing vs sitting), a meta-analysis of more than 50 single studies found no significant influence of body position (supine vs others) on HRV measures, although that included studies which revealed large differences in HRV parameters between sitting and standing positions.^63^ However, in our sample, autonomic activation in the form of HF fell to almost similar levels in teaching (mostly standing and walking) and the task under time pressure (seated).

Against our assumptions stated in Hypothesis 2, we did not find increasing stress in general over time. Perceived stress across groups was meaningfully elevated throughout the observation period compared with reference PSS values.^54^ Unlike Christ,^48^ we did not find increased perceived stress from t_2_ to t_3_, that is, after the beginning of fully independent teaching. This implies that our observation period apparently covered the inverted trough or plateau only, and it did not include the baseline and the ascent of the previously reported, inverted *U*-shape.

The results concerning physiological stress parameters imply that the physiological strain was highest in the very beginning of teaching exposure and receded in the course of teacher training, probably due to trainee teachers gaining more experience. The effect of the experimental situation at the first measurement time point has to be considered a marginal stressor. Participants were used to continuous observation by their mentors in this period and reported that they had not been aware of the chest belt especially during teaching, which was the interval with the largest differences between the first and the other testing time points. We are therefore confident to having measured the stress level caused by the professional situation and not caused by the testing circumstances. Further qualifying the level of physiological strain, physical work, which causes the HR to rise over 130 bpm for continued periods of time, is reported to result in adverse health effects.^21,64^ The limit for permanent strain is considered around 105–100 bpm. In our sample, at t_1_, the mean HR during the teaching activity was slightly above this limit of permanent strain, implying that a considerable number of participants surpassed it; several individuals even surpassed the lesson average of 130 bpm. At t_2_ and t_3_, the mean teaching HR decreased slightly below the lower boundary of the permanent strain limit. Although it is not the first teaching experience in a young teacher’s life (all German student teachers have to complete a practicum for the duration of 1 semester during their studies), our results indicate the reality shock in the beginning of teaching as a significant stress factor in the career of teachers, which is physiologically detectable.

During the final resting interval, HRV was relatively stable across all 3 measurement time points. This indicates that these measures appear to represent a good baseline condition at t_1_. Additionally, this shows that in the resting condition, in contrast to the cardiovascular activation of the HR, the parasympathetic activation is not much affected by either the initial stress of teaching or the subsequent relaxation by becoming accustomed to teaching. Based on the reference values,^34,65^ our participants reached average baseline values, considering young females’ (the majority of our sample) higher parasympathetic activation compared to males and older persons in general. This leads to the conclusion that parasympathetic functioning at the baseline is not (or not much) affected in the initial phase of teacher training.

The influence of the participants’ age on physiological outcomes was completely in line with previous research findings^23,64^ and rather weak, reflecting the relative homogeneity of the trainee teacher group in this respect. The influence of gender was more complex. While higher HR is reported for females,^64^ this was only the case (and not statistically significant) during the teaching interval in our study. In line with previous research^63^ we found higher values of parasympathetic activation among females. However, this difference almost disappeared at the teaching interval. Consequently, the differences between the teaching interval and the resting intervals were larger for females, which might be interpreted as a stronger physiological reactivity of females toward stressful situations. Taking into consideration the participants’ gender might shed further light on mixed findings, such as those reported by Allen et al.;^66^ according to them, in an all-male sample study, no effect on vagal tone was found, while a mixed-gender study showed an effect. However, analysing the data of this study with the female subset only did not yield significantly different results.

In contrast to our assumption in Hypothesis 3 and to previous research in more laboratory-like settings, the physiological parameters showed no significant advantages for the IG, except for HR in the task under time pressure (i3) interval at t_2_ and t_3_, where the IG had significantly higher pre-values, however. The analysis of the relationship between the pre–post change in PSS and biophysiological parameters failed to support the expected changes.

The reduction of perceived stress in the IG supports Hypothesis 3 with regard to the self-reported variable. The development of reduced biophysiological stress and maintained perceived stress in the CG seems paradoxical and might put into perspective findings from other studies on sustained self-reported stress. Concerning the inverted *U*-shape, this implies a possible amelioration of the *U*-shape to a *V*-shape in the mindfulness-trained IG, thereby shortening the extremely stressful phase.

Altogether, the mindfulness intervention proved effective on the level of beliefs (perceived stress). On the physiological level, only existing pre-disadvantages could be offset, but no additional beneficial effects could be demonstrated in this study. In light of some participants’ critically elevated HR levels, this can possibly mean a reduced risk of related diseases for especially vulnerable individuals. More importantly, the reduced perceived stress levels at least indicate improved well-being, even under the inevitable strain, and a greater psychological capacity for effectively dealing with occupational and learning demands.

### Limitations

The present sample consists of trainee teachers who deliberately chose this training among a set of other options. Therefore, the sample might include persons with specific characteristics, such as a stronger interest in stress reduction and personality development or a greater openness compared with other trainees. On one hand, this seems to reduce the generalizability of the results. On the other hand, the motivation to undergo training in mindfulness is a prerequisite for the effects of such an intervention, and the general stress situation found in this sample does not seem implausible on the background of known norm values and research of this phase of the teacher’s career.

An MBI for stress reduction aims to increase the awareness for feelings of increasing stress and the need to relax. Our research design, including pre-set intervals of teaching, rest, and cognitive tasks, tested the biophysiological ability to recover after class during a particularly demanding occupational phase, in which trainee teachers are constantly subject to monitoring and evaluation. On a behavioural level, whether the trainee teachers would actually take breaks to recover after class without external regulation is an open question. This empirical question could be investigated in a follow-up study regarding trainee teachers’ recess habits.

The CG was not active in the sense of participating in another stress-reducing intervention before t_2_. This could induce a performance bias regarding self-reported stress in favour of the IG who might have developed expectations of stress reduction at t_2_. However, this performance bias was attenuated by the fact that CG participants had group support in their regular teacher training courses and were gaining experience in their work which could have led to expectations of a better ability to cope with work demands.

There is an ongoing debate about the need to adjust HRV routinely for respiration. While some researchers believe it is necessary, others hold that respiration and HRV, in particular its parasympathetic influence, have a common origin and that removing respiration-related variance would erroneously remove some of this common influence.^67^ This latter view seems to be more consistent with an understanding of mindfulness as not, at first, changing the respiration rate and then, through this, the parasympathetic activation, but rather as having multiple, interrelated effects on cognition, hormones, respiration, parasympathetic activation, etc., often with cognition as a common antecedent.^15^ In this study, which did not include periods of paced breathing, we did not measure respiratory rates. Therefore, it was not possible to correct for this potentially confounding factor in our statistical models. Instead, we addressed this limitation by analyzing one HRV parameter, i.e., HF, that largely corresponds to the influence of the respiratory cycle and another HRV parameter, i.e., RMSSD, that is relatively free of respiratory influence.^58^ A comparison between the results of these 2 measures showed no major differences overall. Additionally, we visually observed the power spectrum to check whether there were signs of a considerably slower breathing such as in meditators during meditation or athletes at rest that would conflict with the conventional interpretation of the HF band of 0.15 to 0.40 Hz, without finding such signs. Future research should, nevertheless, include the control for respiration even during non-controlled or especially during speech-influenced breathing as during our teaching interval.

The theoretical exposition and presentation of the results might suggest a linear relation between physiological measures and well-being, health, or occupational performance, resulting in the goal of lowering HR to the minimum and boosting HRV to the maximum. To counter this too simplistic understanding, we emphasize the need for basic cardiac activation of living beings. Especially during work situations, the body is not necessarily best adapted to challenges with a maximum parasympathetic activation. This is illustrated by the findings that an adequate challenge and physiological arousal are associated with the feeling of flow^68^ or of moderately elevated HR and attenuated HRV during school and leisure time, which are reported to be correlated with stronger self-efficacy belief,^69^ which in turn is an indicator of various desirable characteristics of successful professionalization of teachers.^70^

### Future Directions

This study demonstrated a strong physiological, probably stress-induced effect in the beginning of teacher training. More testing time points before teacher induction and during the first half year could address the question of when this period of extreme stress on the cardiac system begins and how long it lasts. A longer follow-up could further provide a frame of reference for the interpretation of the stress level during teacher training. A longer follow-up could also determine whether a mindfulness intervention would eventually (in the very long term) have a value-added effect on physiological stress.

The sample size of the present study was rather small underscoring the pilot-character of the present study. To facilitate more sophisticated analyses, such as comparing the change in perceived stress with the change in HRV measures, and increase sensitivity to small effects within the current design, future studies should aim for larger sample sizes.

In this study, the extent of engagement in home practice in the IG was not linked to the training outcomes. Better methods to measure home practice than retrospect self-reports, such as regular logs, are necessary, although to date, evidence regarding this covariate remains inconclusive.^71^ This could shed further light on the importance of regular home practice for stress reduction in MBIs, in particular during the time-demanding phase at the start of teachers’ careers.

## Conclusion

This study investigated the stress of trainee teachers during their first-year induction from the combined perspective of manifest physiological and self-reported data for the first time. The results indicate that the teaching activity poses a challenge to the physiological system of trainee teachers. The stress is particularly visible in the physiological data at the time when trainee teachers have started their teaching experience, about 3 months into their training program. Compared with references from physical occupational activity, the measured level of physiological strain must be considered above the level that is healthy in the long run.

This study did not establish general beneficial effects of the mindfulness intervention on biophysiological parameters, as the intervention group and waiting control group recovered to a similar level at the second testing time point 6 months into teacher training. This study only found that a comparatively higher HR during teaching time and stressful cognitive tasks could be successfully attenuated in the intervention group. Therefore, trainee teachers with critical biophysiological prerequisites might benefit from the intervention on a physiological level in the first year of teacher training. However, the intervention could decrease the perceived stress of participants directly after the intervention and 5 months later, when trainee teachers are at the stage of independent teaching. This might imply beneficial effects on mental health, which could possibly lower the incidence of early attrition and burnout of teachers in the long run.

## Supplemental Material

Supplemental Material - Heart Rate Variability and Perceived Stress in Teacher Training: Facing the Reality Shock With Mindfulness? Click here for additional data file. Supplemental Material for Heart Rate Variability and Perceived Stress in Teacher Training: Facing the Reality Shock With Mindfulness? by Philipp Beuchel and Colin Cramer in Global Advances in Integrative Medicine and Health
