# Supplementary material for: Heart Rate Variability and Perceived Stress in Teacher Training: Facing the Reality Shock With Mindfulness?
Source: Glob Adv Integr Med Health. 2023 May 18;12:27536130231176538. doi: 10.1177/27536130231176538 (PMC10196549; doi:10.1177/27536130231176538)
Supplement: Supplemental Material - Heart Rate Variability and Perceived Stress in Teacher Training: Facing the Reality Shock With Mindfulness? [file sj-pdf-1-gam-10.1177_27536130231176538.pdf]

Supplementary materials

Table 1a. Descriptive statistics for original (non-normalized) RMSSD and HF values at the five intervals: means and *SD*s. (corresponds to Table 1 of the main article)

Interval	Overall			Intervention group			Control group		
	t_1	t_2	t_3	t_1	t_2	t_3	t_1	t_2	t_3
RMSSD									
1	19.0 (10.3)	21.6 (8.9)	24.2 (11.1)	16.4 (5.6)	20.6 (7.6)	21.9 (9.6)	21.3 (12.7)	22.4 (9.9)	26.0 (12.0)
2	39.5 (32.0)	45.2 (25.8)	45.4(29.2)	32.3 (20.9)	36.5 (20.6)	37.3 (20.6)	45.7 (38.7)	52.7 (27.8)	51.8 (33.7)
3	30.6 (30.3)	32.6 (22.1)	38.5 (33.6)	21.7 (12.6)	26.8 (15.7)	31.1 (21.5)	38.6 (38.7)	37.3 (25.6)	44.7 (40.7)
4	37.3 (31.9)	38.0 (23.9)	41.5 (25.8)	30.2 (21.2)	32.0 (13.5)	32.8 (15.4)	43.4 (38.2)	42.9 (29.3)	48.9 (30.6)
5	43.7 (33.6)	43.2 (28.0)	48.0 (32.3)	38.5 (29.8)	35.8 (19.2)	43.8 (30.6)	48.3 (36.7)	49.2 (32.7)	51.4 (34.1)
HF									
1	223 (194)	242 (209)	309 (288)	198 (126)	213 (153)	230 (162)	250 (240)	266 (245)	371 (350)
2	1001 (1571)	1052 (1182)	1122 (1563)	614 (650)	762 (1045)	709 (846)	1234 (1969)	1236 (1237)	1453 (1918)
3	771 (2018)	456 (651)	748 (1561)	222 (194)	268 (329)	434 (532)	1167 (2631)	585 (784)	1012 (2051)
4	1051 (1947)	748 (896)	860 (1157)	519 (692)	433 (369)	472 (507)	1394 (2449)	962 (1093)	1187 (1437)
5	1159 (1662)	1046 (1427)	1071 (1285)	895 (1467)	643 (884)	893 (1141)	1255 (1756)	1302 (1677)	1212 (1402)

Table 1b. Descriptive statistics for LF, lnLF and LF/HF ratio at the five intervals: means and *SD*s.

Interval	Overall			Intervention group			Control group		
	t_1	t_2	t_3	t_1	t_2	t_3	t_1	t_2	t_3
LF									
1	1031 (611)	1121 (611)	1405 (915)	1076 (528)	1086 (412)	1299 (849)	998 (679)	1147 (729)	1490 (970)
2	1837 (2609)	2278 (2589)	1728 (2143)	1617 (2367)	2394 (3254)	1578 (2130)	2023 (2811)	2207 (2031)	1870 (2165)
3	741 (829)	627 (725)	833 (1300)	684 (828)	601 (808)	883 (1625)	789 (844)	650 (680)	792 (995)
4	1190 (1283)	1113 (1286)	1046 (1173)	963 (816)	999 (662)	776 (484)	1391 (1539)	1228 (1603)	1291 (1480)
5	2487 (3209)	2124 (2701)	2106 (2876)	2068 (2387)	1901 (2316)	2095 (3345)	2849 (3726)	2339 (2980)	2146 (2516)
lnLF									
1	6.76 (0.69)	6.92 (0.47)	7.08 (0.58)	6.89 (0.48)	6.94 (0.35)	6.99 (0.63)	6.66 (0.81)	6.89 (0.55)	7.15 (0.54)
2	7.01 (1.05)	7.37 (0.91)	7.07 (0.95)	6.95 (0.94)	7.37 (0.91)	6.99 (0.94)	7.06 (1.14)	7.37 (0.93)	7.14 (0.95)
3	6.17 (0.91)	6.01 (1.09)	6.19 (0.96)	6.11 (0.91)	6.07 (0.84)	6.20 (0.95)	6.23 (0.93)	5.97 (1.26)	6.19 (0.99)
4	6.70 (0.85)	6.57 (1.06)	6.56 (0.91)	6.65 (0.62)	6.70 (0.70)	6.46 (0.71)	6.74 (1.01)	6.49 (1.27)	6.66 (1.04)
5	7.35 (0.98)	7.19 (1.10)	7.23 (0.99)	7.36 (0.74)	7.19 (0.91)	7.20 (0.91)	7.34 (1.15)	7.19 (1.24)	7.24 (1.07)
LF/HF									

1	6.35 (2.81)	6.55 (3.02)	6.56 (3.35)	6.54 (2.76)	6.57 (2.44)	6.47 (2.49)	6.20 (2.90)	6.53 (3.45)	6.63 (3.97)
2	3.90 (2.58)	3.87 (2.83)	3.88 (4.16)	3.89 (2.70)	4.53 (2.72)	3.61 (4.24)	3.91 (2.54)	3.37 (2.87)	4.10 (4.19)
3	4.56 (4.41)	3.45 (3.09)	3.11 (4.05)	4.71 (5.07)	4.11 (3.45)	3.19 (3.15)	4.45 (3.36)	2.98 (2.81)	3.04 (4.76)
4	3.88 (4.63)	3.04 (3.04)	2.82 (3.50)	4.18 (5.87)	3.32 (2.38)	3.55 (4.53)	3.64 (3.52)	2.83 (3.48)	2.20 (2.29)
5	4.84 (3.69)	4.12 (4.03)	4.03 (3.80)	5.21 (3.80)	5.38 (5.21)	4.48 (5.04)	4.56 (3.66)	3.20 (2.67)	3.67 (2.50)

Table 2. Descriptive statistics for original (non-normalized) RMSSD and HF values in the two age and gender groups at the five intervals: means and *SD*s. (corresponds to Table 4 of the main article)

	Interval	Mean (*SD*)	
Age		Younger	Older
RMSSD	1	20.2 (11.0)	20.4 (9.2)
	2	39.9 (29.1)	35.9 (28.8)
	3	30.8 (33.0)	28.3 (23.0)
	4	39.3 (29.0)	31.7 (24.3)
	5	45.2 (33.2)	37.2 (27.9)
HF	1	228 (243)	226 (220)
	2	915 (1540)	679 (1234)
	3	586 (1908)	373 (729)
	4	847 (1644)	563 (959)
	5	975 (1548)	787 (1295)
Gender		Female	Male
RMSSD	1	22.5 (10.7)	18.1 (8.1)
	2	47.5 (30.8)	28.4 (15.4)
	3	36.8 (32.0)	22.4 (13.3)
	4	40.7 (28.6)	30.3 (20.8)
	5	47.5 (32.8)	34.9 (23.5)
HF	1	277 (252)	177 (146)
	2	1198 (1589)	396 (346)
	3	700 (1741)	259 (251)
	4	911 (1562)	498 (663)
	5	1209 (1583)	553 (793)

Notes: Younger stands for participants aged < 28 years (= median).
